# Respiratory dysfunction in whiplash associated disorders (WAD) with cervical plexus syndrome – A case report

**DOI:** 10.1016/j.sipas.2025.100271

**Published:** 2025-01-12

**Authors:** NA Nystrom, SR Daulat, A Zakaria, M Petersen, VM Moodley, LP. Champagne

**Affiliations:** a University of Stavanger Medical Center, Department of Hand- and Plastic Surgery, Stavanger, Norway; b Clinique Bellevue, Muségate 18, Stavanger, Norway; c University of Pittsburgh School of Medicine, Pittsburgh, PA, USA; d Arizona Center for Hand to Shoulder Surgery, Phoenix, AZ, USA; e Department of Respiratory Medicine, University of Stavanger Medical Center, Stavanger, Norway; f Department of Orthopaedic Surgery, University of Arizona, Phoenix, AZ, USA

**Keywords:** Whiplash, Cervical plexus syndrome, Pain, Respiratory dysfunction, Staccato speech, Fluency disorder, Dysarthria, Peripheral nerve surgery

## Abstract

Whiplash Associated Disorders (WAD) represents a chronic post-traumatic pain syndrome from indirect flexion-extension trauma to the neck. The condition exhibits significant variability among affected individuals and can involve numerous secondary symptoms, including but not limited to myalgia, central sensitization, migraines, photophobia, jaw pain, dysphagia, joint stiffness, and tinnitus, while significant breathing problems are not commonly associated with or prominently considered in WAD.

Herein, we present the diagnosis and successful surgical treatment of severe respiratory dysfunction and staccato speech in a patient with WAD, who over a period of more than ten years underwent multiple spirometry evaluations for breathing difficulties that correlated with the severity of neck pain. In 2019, his condition deteriorated, with significantly increased pain and dyspnea leading to further evaluations that included laboratory studies and consultations with specialists in neurosurgery, neurology, pulmonology, neurophysiology, ENT, general internal medicine, cardiology, radiology, speech pathology, physical medicine, orthopedic surgery, and hand surgery at three separate academic centers in Norway.

Eventually, the patient was diagnosed with a condition that is regularly observed among patients referred to our office for evaluation and surgical treatment of chronic, whiplash related pain, and that we propose to label Cervical Plexus Syndrome.

One year following exploration and neurolysis of sensory nerves to the right and left superficial cervical plexus, the patient remains pain free, with unimpeded speech and breathing as demonstrated by postoperative spirometry and video recordings.

Although the underlying pathophysiology remains unclear, we report what we believe to be the first successful surgical treatment of serious respiratory dysfunction from pain generators in tissue that historically is considered anatomically and functionally separate from the mechanics of breathing. Further investigation will be needed to determine prevalence of respiratory dysfunction in chronic neck pain.

## Introduction

Chronic whiplash, sometimes referred to as Whiplash Associated Disorders (WAD), is a loosely defined chronic pain syndrome, triggered by flexion-extension, or “whiplash” trauma to the neck [1]. While clinical presentations of WAD vary across a broad spectrum of symptoms, limited success rates with non-invasive treatment modalities[2] and a general paucity of positive radiological or electrodiagnostic findings frustrate clinicians’ diagnostic and therapeutic efforts[3,4] and increase the risk of ineffective interventions that may expose patients to social, medical and emotional hazards including heroic or harmful treatment and the harm of questionable psychiatric explanatory models [[5], [6], [7]].

While functional limitations are near universal in WAD, empirical data confirm that loss of neck and/or shoulder mobility is not necessarily attributable to joint pathology, primary muscle weakness, or direct nerve damage, but more frequently to pain inhibition from seemingly unrelated structures [[8], [9], [10], [11], [12]].

Recent studies have also emphasized the role of central sensitization, which by definition entails generalized lowering of peripheral pain thresholds, as potential explanation for certain aspects of persistent pain in WAD [9,10,13]. Moreover, a significant overlap between chronic pain and other systemic dysfunction, including side effects of prescription drugs, secondary cognitive problems, and impaired sleep, vision, or swallowing, further complicate the effort to establish appropriate management strategies [4].

Although respiratory dysfunction has been described in association with chronic whiplash [14], past literature does not provide a valid explanatory model or an effective treatment strategy in the absence of demonstrable injury to neuromuscular structures directly involved with respiratory function [[15], [16], [17], [18], [19], [20], [21]]. The present case history suggests sensory nerve entrapment as a potentially treatable cause for inhibition of seemingly unrelated motor functions including but not limited to respiration.

## Patient history

### Neck pain of unclear origin

In 1992, a previously healthy man born 1952 contacted his primary care physician with a several months history of neck pain and headaches without any clear history of a precipitating trauma. Over time, the condition became increasingly disabling while remaining refractory to conservative treatment modalities that included physical therapy and a variety of prescription drugs.

#### 1st spine surgery

In 2001, nine years following the debut of symptoms, the patient was diagnosed with cervical spondylosis for which he underwent foraminotomy for root decompression across three cervical segments (C4–5–7). Since all surgical treatment options were believed to have been exhausted when the procedure did not alleviate or worsen any of his symptoms, the patient was instead referred to pain management.

#### Pain worse, restricted breathing after trauma

Following a fall against an outstretched arm in 2005, the patient's condition worsened to include left arm weakness, increased neck pain, and dyspnea/chest pain, which hitherto had not been described in any medical records.

#### Whiplash; pain worse

Although spirometry, chest radiography and fluoroscopy findings in January 2009 showed a restrictive respiratory pattern with hypomobility of the diaphragm (Table 1), continued investigation including EMG and nerve conduction studies did not provide a valid explanatory model for the breathing problem that debuted after a trauma that is likely to have included flexion-extension or abduction strain to supporting soft tissues of the cervical spine. Later that same year, after exposure to further indirect neck trauma in a motor vehicle crash, his condition deteriorated further.

**Table 1:** Spirometry data 2009–2024.

#### 2nd spine surgery

Because of increased neck pain, troubling shortness of breath, and further radiological progress of cervical spondylosis but still no electrodiagnostic evidence of neuromuscular dysfunction, the patient underwent a second cervical spine procedure (C7-T1 segment decompression & disk replacement) in January 2010. As documented in medical records, the breathing problems persisted post op. Table I.

#### Chronic trapezius myalgia

In addition to the more disabling pain and respiratory dysfunction, the patient suffered from diffuse upper extremity numbness and paresthesias that in 2016 prompted a referral for consultation with a specialist in hand surgery. While the clinical findings did not suggest an affliction of the brachial plexus (C5-Th1) or any of its peripheral branches, the patient was diagnosed with bilateral chronic trapezius compartment syndrome, a condition that generally responds well to surgical treatment. Unlike musculature in the upper and lower extremities, however, the size and segmentation of the trapezius makes it virtually impossible in clinical practice to perform a complete fasciotomy as one single procedure. It is therefore not unusual that treatment for chronic trapezius myalgia requires more than one operation.

#### Fasciectomy, nerve decompression (2 procedures)

After a bilateral, partial trapezius fasciectomy, which was performed through a dorsal midline incision, the patient experienced less pain in his right shoulder girdle, improved albeit not fully normalized respiratory function, but still troubling left sided symptoms. Table I.

In May 2018, the patient returned to the operating room for an extended left trapezius fasciectomy and neurolysis of the ipsilateral spinal accessory nerve, a predominantly efferent cranial nerve to the trapezius and sterno-cleido-mastoid (SCM) muscles [8]

With near complete freedom of shoulder-neck pain and normalized spirometry readings post op (Table I), the patient discontinued all prescription pain killers and returned to work as a truck driver after more than five years on full-time disability.

#### New and worsened symptoms

The patient continued to enjoy essentially unimpeded respiration until 2021, when he for reasons unknown developed intense left sided temporal headaches and pain radiating from the left side of his neck across the left shoulder and upper left thorax in a pattern described as different from before the 2016 and 2017 surgeries. With increased pain, breathing problems returned to the point of frustrating even minor physical exertion including speech.

#### Cervical plexus syndrome

In April 2022, increasingly severe ache, numbness and paresthesias in the patient's left upper extremity prompted a referral for evaluation by a specialist in hand surgery. While an affliction of the brachial plexus or its peripheral branches was again excluded based on clinical exam, diagnostic right and left cervical plexus blocks[22] resulted in freedom of pain and upper extremity paresthesias, improved neck and shoulder mobility, and normalized speech/respiration for the duration of the anesthetic. Table 1. Video 1.

Although the patient met our criteria for surgery, definitive treatment was postponed when coverage for the procedure was denied by the Norwegian Health Economics Administration (Helfo). The clinical investigation instead continued with referrals to multiple specialists in neurology, neurosurgery, ENT, internal medicine, general practice, pain medicine, physical therapy, and speech pathology at three different academic centers in Norway, where clinical and laboratory findings including radiology, electrophysiology, polysomnography, spirometry, and direct laryngoscopy did provide evidence of sleep apnea and hypo-mobility of the diaphragm but no explanation or treatment plan.

Over a total of 18 months, the patient was seen in the hand surgery office on twelve occasions. At each visit, cervical plexus blocks confirmed cervical plexus syndrome as a valid explanatory model for neck pain and respiratory dysfunction by providing total or near total, albeit temporary, relief of all symptoms.

As the patient's condition continued to worsen, with predominantly left sided symptoms including upper thoracic pain that on three separate occasions prompted hospitalization for cardiac monitoring, he became increasingly exhausted, socially isolated, and depressed.

## Anatomical considerations

The cervical plexus (C1–4) has traditionally been described in terms of a deep and a superficial portion, where “superficial” refers to a group of sensory nerves without known functional relation to respiration: the greater auricular, lesser occipital, transverse cervical, and supraclavicular nerves [22]. In contrast, “deep” refers to a collection of efferent structures including a significant contribution to the phrenic nerve (C3–5).

While the efferent portions of the cervical plexus are mostly covered by the SCM, the four sensory nerves emerge around the posterior aspect of the muscle for trajectories that are mostly subcutaneous.

## Neurolysis of (superficial) cervical plexus

In December 2023, the patient returned to the operating room for exploration and neurolysis of right and left cervical plexus. At that time, repeated preoperative diagnostic injections with local anesthetic had consistently resulted in improved respiratory function and adequate distribution of surface anesthesia without any evidence of motor block. Based on that observation, the surgical dissection was limited to the superficial, sensory components of the cervical plexus.

**Fig. 1:**
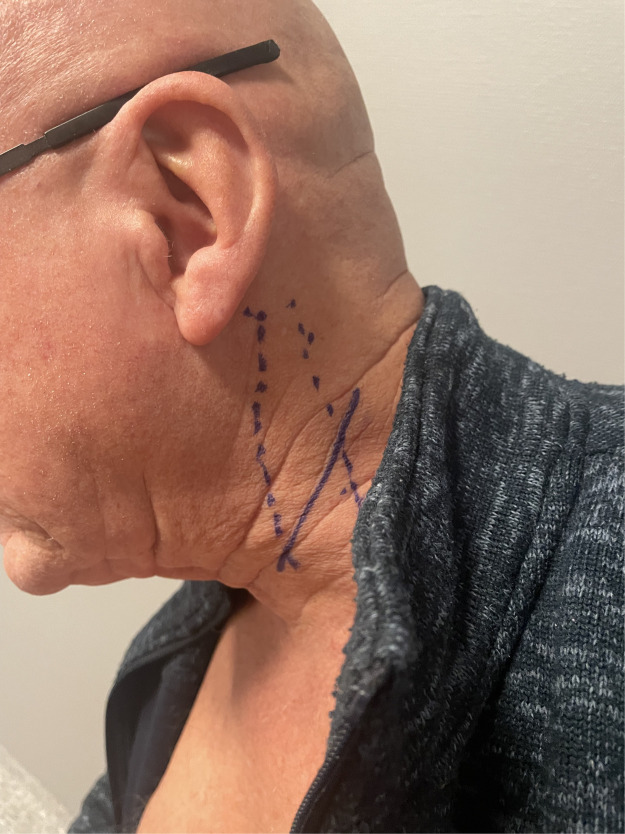
Preoperative outline of (left) sterno-cleido-mastoid muscle and skin incision centered over Erb's point.

The operation was performed through a skin incision as outlined in Fig. 1, under partial local anesthesia and intermittent IV sedation according to an established routine that allows patients to participate actively and provide real-time feedback during vital portions of the procedure [8,11,12]. The intraoperative findings were essentially unremarkable but did confirm a close relation between nerve and muscle fascia, requiring meticulous dissection under loupe magnification for adequate neurolysis/decompression.

## Outcome of treatment

Significant functional improvement was noticed during and immediately after surgery, with near complete relief of all preoperative symptoms including those pertaining to speech and respiration. Video 2

Four months post op the patient described himself as active and for all intents and purposes unrestricted by pain or shortness of breath, with unimpeded respiration as confirmed by spirometry. Table 1.

Nine months post op, the patient has discontinued all use of prescription or over-the-counter pain medication, and only occasionally needs CPAP for restful sleep. While spirometry again confirmed respiratory function within normal limits for the patient's age, body plethysmography confirmed normal findings without any evidence of restrictive or obstructive lung disease.

One year post surgery, the patient describes himself as pain free and physically active without any speech or breathing impediment. Interestingly, he also volunteers that he since the day of surgery has been free of the hitherto ever present left sided chest pain that on three occasions had prompted acute admission and in-house monitoring for suspect myocardial infarct. Video 3.

## Discussion

Whiplash Associated Disorders (WAD) is an umbrella diagnosis that covers a broad and variegated spectrum of chronic symptoms precipitated by acute indirect flexion-extension trauma to the neck [1]. Since symptomatic lesions are not always possible to identify with imaging or electrodiagnostic techniques in WAD, a true anatomical diagnosis - a prerequisite for successful surgical treatment - may depend entirely on the accuracy of physical examination including diagnostic blocks to identify focal origin(s) of any symptom [3,[8], [9], [10], [11], [12]].

Neck pain has previously been reported as potential cause for respiratory dysfunction with potentially more than one valid explanatory model [[14], [15], [16], [17], [18], [19], [20]]. In that context, it is not without clinical significance if a distant lesion can be identified as root cause of symptoms and potentially provide a treatment plan.

There is no indication that any diagnostic injection or surgery that our patient underwent involved the deep cervical plexus, specifically the phrenic nerve, which allows us to conclude that his respiratory problems were solely an expression of pain inhibition from the SCM/cervical plexus complex. That model also explains past failures to establish a successful treatment strategy based on imaging or electrodiagnostic studies.

The clinical entity that we label Cervical Plexus Syndrome (CPS) is, in our experience, a predominantly posttraumatic phenomenon most frequently diagnosed and treated among patients who seek medical attention for chronic neck pain and a history of whiplash. Although the condition is not uncommon, dyspnea or speech impediment has rarely – if ever – been spontaneously reported by our patients or mentioned in their medical records. In fact, we are not aware of any previously published example of significant respiratory inhibition from injury to tissues that traditionally are considered anatomically and functionally unrelated to the diaphragm and phrenic nerve.

The conclusion that our patient's breathing problem was due to distant pain inhibition is supported by several observations:

– The severity of respiratory symptoms varied with the intensity of neck- and shoulder pain.
– Diagnostic injections of local anesthetic resulted in temporarily normalized speech and breathing.
– No major efferent nerves from or near the cervical plexus, e.g. the phrenic nerve (C3–5), the spinal accessory nerve (CN XI), or the ansa cervicalis (C1-C3) were exposed or manipulated during surgery.
– Speech and respiratory function have remained within normal limits since the day of surgery.
– Chest pain, in the past interpreted and treated as angina pectoris, is with the patient's words “just gone” since the day of surgery, which is suggestive of a preoperative entrapment neuropathy involving the left supraclavicular nerve, a branch of the superficial cervical plexus.

Pain inhibition from entrapment of dedicated sensory nerves is not the only valid explanatory model for our patient's dyspnea since chronic compartment syndrome of the SCM, an accessory respiratory muscle, must be accepted as an alternative or contributing causative factor.

Competing explanatory models, although not without interest in other contexts, are likely irrelevant in this case since the anatomical relationship between SCM and the cervical plexus is so close that fasciotomy and thus *de facto* treatment of a compartment syndrome (if one exists) would always and by necessity be an integrated part of the neurolysis.

Although we interpret our patient's history as a confirmation of cervical plexus syndrome as the main underlying cause for his severe dyspnea, we do exclude that other distant pain generators may have similar inhibitory effect on respiration since his lung function improved temporarily after surgical treatment of chronic trapezius myalgia.

Twelve months post op, the patient remains free of the previously constant left thoracic pain that on three occasions had prompted inpatient monitoring for suspected myocardial infarct. In retrospect, he has described a preoperative pattern of chest pain that closely corresponds to the distribution of the supraclavicular nerve, a sensory component of the cervical plexus.

That we previously have not identified severe dyspnea as part of the chronic whiplash syndrome may suggest a rare relationship, but we also do not exclude that we in the past may have simply ignored a potentially causative correlation between pain and respiratory dysfunction among patients seeking help for unremitting .

## Conclusion

This case underscores the importance of an integrated approach across specialty lines in WAD but does not allow any conclusion regarding the prevalence of surgically treatable respiratory dysfunction due to pain inhibition from distant structures. We have for that reason updated our clinical routines to include questions pertaining to speech and breathing, adding pre- and postoperative spirometry to our standard protocol for patients who confirm speech impediment or shortness of breath since exposure to whiplash trauma.

## CRediT authorship contribution statement

**NA Nystrom:** Conceptualization, Formal analysis, Investigation, Methodology, Validation, Writing – original draft, Writing – review & editing. **SR Daulat:** Writing – review & editing, Writing – original draft, Software. **A Zakaria:** Writing – review & editing, Data curation. **M Petersen:** Data curation, Writing – original draft. **VM Moodley:** Software. **LP. Champagne:** Resources, Writing – original draft.

## Declaration of competing interest

The authors declare that they have no known competing financial interests or personal relationships that could have appeared to influence the work reported in this paper.

## Appendix Supplementary materials

**Video 1** Speech, forced expiration, and shoulder mobility before and after bilateral diagnostic block of the superficial cervical plexus. Although the patient communicates in his native language, the effect of the block on the *pattern* of speech is recognizable also for non-Norwegian speakers. Image, video 1

**Video 2** Speech, forced expiration, and shoulder mobility before and immediately following bilateral exploration and neurolysis of the (superficial) cervical plexus. Image, video 2

**Video 3** One year post surgery, the patient runs without pain or respiratory problems. Image, video 3

## References

[1] Spitzer W.O., Skovron M.L., Salmi L.R. (1995). Scientific monograph of the quebec task force on whiplash-associated disorders: redefining “whiplash” and its management. Spine.

[2] Verhagen A.P., Scholten-Peeters G.G.G.M., van Wijngaarden S., de Bie R.A., SMA Bierma-Zeinstra (2007). Conservative treatments for whiplash. Cochrane Database Syst Rev.

[3] Curatolo M., Bogduk N., Ivancic P.C., McLean S.A., Siegmund G.P., Winkelstein B.A. (2011). The role of tissue damage in whiplash-associated disorders: discussion paper 1. Spine.

[4] Bring G. (2000). Whiplash-associated injuries and disorders : biomedical aspects of a multifaceted problem. Arbetslivsinstitutet.

[5] Ferrari R., Kwan O., Russell A.S., Pearce J.M., Schrader H. (1999). The best approach to the problem of whiplash? One ticket to Lithuania, please. Clin Exp Rheumatol.

[6] Cassidy J.D., Carroll L.J., Côté P., Lemstra M., Berglund A., Nygren A. (2000). Effect of eliminating compensation for pain and suffering on the outcome of insurance claims for whiplash injury. N Engl J Med.

[7] Volle E., Montazem A. (2001). MRI video diagnosis and surgical therapy of soft tissue trauma to the craniocervical junction. Ear Nose Throat J.

[8] Nystrom N.A., Champagne L.P., Freeman M., Blix E. (2010). Surgical fasciectomy of the trapezius muscle combined with neurolysis of the Spinal accessory nerve; results and long-term follow-up in 30 consecutive cases of refractory chronic whiplash syndrome. J Brachial Plex Peripher Nerve Inj.

[9] Freeman M.D., Nystrom A., Centeno C. (2009). Chronic whiplash and central sensitization; an evaluation of the role of a myofascial trigger points in pain modulation. J Brachial Plex Peripher Nerve Inj.

[10] Nystrom N.A., Freeman M.D. (2018). Central sensitization is modulated following trigger point anesthetization in patients with chronic pain from whiplash trauma. A double-blind, placebo-controlled, crossover study. Pain Med.

[11] Duffy M.F., Stuberg W., DeJong S., Gold K.V., Nystrom N.A. (2004). Case report: whiplash-associated disorder from a low-velocity bumper car collision: history, evaluation, and surgery. Spine.

[12] Nystrom A., Ginsburg G.M., Stuberg W., Dejong S. (2009). Pre- and post-operative gait analysis for evaluation of neck pain in chronic whiplash. J Brachial Plex Peripher Nerve Inj.

[13] Van Oosterwijck J., Nijs J., Meeus M., Paul L. (2013). Evidence for central sensitization in chronic whiplash: a systematic literature review. Eur J Pain.

[14] Edwards I.J., Lall V.K., Paton J.F. (2015). Neck muscle afferents influence oromotor and cardiorespiratory brainstem neural circuits. Brain Struct Funct.

[15] Nair S.P., Panchabhai C.S., Panhale V. (2022). Chronic neck pain and respiratory dysfunction: a review paper. Bull Fac Phys Ther.

[16] McCool F.D., Tzelepis G.E. (2012). Dysfunction of the diaphragm. N Engl J Med.

[17] Dimitriadis Z., Kapreli E., Strimpakos N., Oldham J. (2013). Respiratory weakness in patients with chronic neck pain. Man Ther.

[18] Kahlaee A.H., Ghamkhar L., Arab A.M. (2017). The association between neck pain and pulmonary function: a systematic review. Am J Phys Med Rehabil.

[19] Sekiguchi H., Minei A., Noborikawa M. (2020). Difference in electromyographic activity between the trapezius muscle and other neck accessory muscles under an increase in inspiratory resistive loading in the supine position. Respir Physiol Neurobiol.

[20] Legrand A., Schneider E., Gevenois P.A., De Troyer A. (2003). Respiratory effects of the scalene and sternomastoid muscles in humans. J Appl Physiol.

[21] Kapreli E., Vourazanis E., Strimpakos N. (2008). Neck pain causes respiratory dysfunction. Med Hypotheses.

[22] Kim J.S., Ko J.S., Bang S., Kim H., SY Lee (2018). Cervical plexus block. Korean J Anesthesiol.

